# Assessment of Potential Anti-Methanogenic and Antimicrobial Activity of Ethyl Nitroacetate, α-Lipoic Acid, Taurine and L-Cysteinesulfinic Acid In Vitro

**DOI:** 10.3390/microorganisms12010034

**Published:** 2023-12-23

**Authors:** Gizem Levent, Aleksandar Božić, Branko T. Petrujkić, Todd R. Callaway, Toni L. Poole, Tawni L. Crippen, Roger B. Harvey, Pedro Ochoa-García, Agustin Corral-Luna, Kathleen M. Yeater, Robin C. Anderson

**Affiliations:** 1 School of Veterinary Medicine, Texas Tech University, Lubbock, TX 79409, USA; gizem.levent@ttu.edu; 2 Faculty of Agriculture, Department of Animal Science, University of Novi Sad, 21000 Novi Sad, Serbia; aleksandar.bozic@stocarstvo.edu.rs; 3 Department of Nutrition and Botany, Faculty of Veterinary Medicine, University of Belgrade, 110000 Belgrade, Serbia; petrujkic@yahoo.com; 4 Department of Animal and Dairy Science, University of Georgia, Athens, GA 30609, USA; todd.callaway@uga.edu; 5 United States Department of Agriculture/Agricultural Research Service, Southern Plains Agricultural Research Center, College Station, TX 77845, USA; toni.poole@usda.gov (T.L.P.); tc.crippen@usda.gov (T.L.C.); roger.harvey@usda.gov (R.B.H.); 6 Facultad de Zootecnia y Ecología, Universidad Autónoma de Chihuahua, Chihuahua 31000, Mexico; peteaog@gmail.com (P.O.-G.); acorral@uach.mx (A.C.-L.); 7 United States Department of Agriculture/Agricultural Research Service, Office of the Area Director, 104 Ambrose Hill, Williamsburg, VA 20250, USA

**Keywords:** ethyl nitroacetate, *Escherichia coli*, α-lipoic acid, rumen methane inhibitors, *Salmonella*

## Abstract

Livestock producers need new technologies to maintain the optimal health and well-being of their animals while minimizing the risks of propagating and disseminating pathogenic and antimicrobial-resistant bacteria to humans or other animals. Where possible, these interventions should contribute to the efficiency and profitability of animal production to avoid passing costs on to consumers. In this study, we examined the potential of nitroethane, 3-nitro-1-propionate, ethyl nitroacetate, taurine and L-cysteinesulfinic acid to modulate rumen methane production, a digestive inefficiency that results in the loss of up to 12% of the host’s dietary energy intake and a major contributor of methane as a greenhouse gas to the atmosphere. The potential for these compounds to inhibit the foodborne pathogens, *Escherichia coli* O157:H7 and *Salmonella* Typhimurium DT104, was also tested. The results from the present study revealed that anaerobically grown O157:H7 and DT104 treated with the methanogenic inhibitor, ethyl nitroacetate, at concentrations of 3 and 9 mM had decreased (*p* < 0.05) mean specific growth rates of O157:H7 (by 22 to 36%) and of DT104 (by 16 to 26%) when compared to controls (0.823 and 0.886 h^−1^, respectively). The growth rates of O157:H7 and DT104 were decreased (*p* < 0.05) from controls by 31 to 73% and by 41 to 78% by α-lipoic acid, which we also found to inhibit in vitro rumen methanogenesis up to 66% (*p* < 0.05). Ethyl nitroacetate was mainly bacteriostatic, whereas 9 mM α-lipoic acid decreased (*p* < 0.05) maximal optical densities (measured at 600 nm) of O157:H7 and DT104 by 25 and 42% compared to controls (0.448 and 0.451, respectively). In the present study, the other oxidized nitro and organosulfur compounds were neither antimicrobial nor anti-methanogenic.

## 1. Introduction

Certain oxidized nitro-containing compounds such as nitrate as well as some short-chain nitroalkanes and oxy-nitrocompounds are potent inhibitors of ruminal methanogenesis, a digestive inefficiency that can result in the loss of up to 15% of dietary intake for forage-fed cattle and 2 to 4% for concentrate fed cattle [1,2]. Some of these nitroalkanes have also been reported to inhibit the growth of important foodborne pathogens such as enterohemorrhagic *Escherichia coli*, *Campylobacter* species *jejuni* and *coli*, *Salmonella enterica* serovar Typhimurium and *Staphylococcus* species *aureus, epidermidis* and *hyicus* [3,4,5,6,7,8,9,10,11]. 

Mechanistically, compounds such as oxy-nitropropanol act as specific inhibitors of the functionally critical methyl CoM reductase enzyme of rumen methanogenic archaea whereas nitrate acts as an alternative anaerobic electron acceptor preferentially allowing the competitive consumption of reducing equivalents at the expense of rumen methanogenesis [12,13]. The mode of action of the other aliphatic nitrocompounds tested is less clear, although many have been implicated in inhibiting the oxidation of hydrogen or formate thereby depriving methanogens of important major reducing substrates for methanogenesis [14]. It has also been reported that in certain cases, such as with nitroethane or the naturally occurring phytotoxins 3-nitro-1-propionate or 3-nitro-1-propanol, these may serve as anaerobic electron acceptors by the ruminal bacterium *Denitrobacterium detoxificans* thereby competing against methanogens for consumption of available reducing substrates [15]. It is unknown, however, whether the nitrocompound-caused inhibition of hydrogen and formate oxidation is a more potent direct effect against methanogens than their potential use as alternative electron acceptors [13,15]. While the mechanistic details regarding the methanogenic-modulating activity of the nitrocompounds have not been fully described, it seems likely that these potential mechanisms could contribute to the inhibition of certain foodborne pathogens. For instance, *E. coli* and *Salmonella* can express hydrogen and formate metabolizing enzymes, that depending on growth conditions, either produce or consume hydrogen or formate [16,17]. Based on early reports that nitroethane is an inhibitor of the hydrogenase activity of *Clostridium pasteurianium* [18], it is reasonable to hypothesize that nitrocompound-caused inhibition of hydrogenase may inhibit the growth of *E. coli* and *Salmonella* although this has yet to be confirmed. In support of this hypothesis, evidence from mouse colonization studies has shown decreased mortality and gut colonization with hydrogenase deficient *Salmonella* [19,20,21].

Certain organosulfur compounds, such as α-lipoic acid and taurine, can function as anaerobic electron acceptors or carriers. Additionally, they or their reduced metabolites exhibit antioxidant and anti-inflamatory activity [22,23]. Thus, it is tempting to hypothesize that oxidized sulfur compounds might potentially function similarly to the oxidized nitrocompounds, either by directly modulating methanogenesis or by consuming electrons for their reduction at the expense of rumen methanogenesis. Moreover, certain organosulfur compounds, including sulfonates and sulfinic acids, such as taurine and L-cysteinesulfinic acid have been reported to serve as anaerobic electron acceptors and antimicrobial agents [24,25,26,27].

To date, only a limited number of different nitro- and organosulfur compounds have been examined as potential methane modulators and more work is needed to identify candidate compounds or mixtures of compounds that are sufficiently potent and selective at inhibiting rumen methane production as well as pathogenic microbes while remaining safe and nonhazardous for practical administration. From a practical perspective, interventions that can inhibit ruminal methane emissions while concurrently decreasing the carriage of foodborne pathogens by disrupting the oral-fecal route of pathogen transmission may provide an economic incentive for livestock producers to adopt such technologies. Accordingly, the objectives of this study were to explore the anti-methanogenic as well as the anti-*E. coli* O157:H7 and anti-*S.* Typhimurium potential of variety of readily available nitro- and organosulfur compounds. Information obtained from this work may ultimately lead to the development of feed additives to help livestock producers produce safer meat and milk while contributing to a cleaner environment.

## 2. Materials and Methods

### 2.1. Comparison of Antimicrobial Effects against Escherichia coli O157:H7 and Salmonella enterica Serovar Typhimurium

*Escherichia coli* O157:H7 strain 933 was obtained from the American Type Culture Collection (Manassas, VA, USA; ATCC 43895) and *Salmonella enterica* serovar Typhimurium DT104 was graciously provided by Dr. Paula Cray when she was affiliated with the USDA/ARS National Animal Disease Center (Ames, IA, USA). The strains were resuscitated from stock cultures frozen at −80 °C in 20% glycerol and each was cultured in Tryptic Soy Broth (Difco, Becton Dickinson, Sparks, MD, USA) buffered to pH 6.7 with 0.1 M sodium phosphate. All cultures were grown in a broth that had been prepared anaerobically under a 100% nitrogen gas phase. The potential modulators were added to incubation tubes (*n* = 3/treatment concentration) of this and following experiments as small volumes (<0.5 mL) of filter sterilized (0.2 µm Millipore, Burlington, MA, USA) stock solutions to achieve 0, 3 or 9 mM. These concentrations were selected to approximate doses used in earlier studies [7,9]. Stock solutions of nitroethane and ethyl nitroacetate were prepared as sodium salts [28] and diluted appropriately with deionized water. Stock solutions of taurine 3-nitro-1-propionic acid and L-cysteinesulfinic acid were prepared in deionized water and α-lipoic acid (DL 6,8 thioctic acid) was prepared in 0.5 M NaOH. All chemicals were purchased from Sigma-Aldrich (St. Louis, MO, USA). Growth was measured by recording absorbance at 600 nm on a Spectronic 20D+ spectrophotometer) Spectronic Instruments, Inc., Rochester, NY, USA) during a 24 h incubation period. Optical densities, presented as change from initial OD600 nm, were used in charts and in calculations of mean specific growth rates, determined during the first 2 to 2.5 h of growth, using (ln OD time 2 − ln OD time 1)/(time 2 − time 1) [29].

### 2.2. Comparison of Effects of Treatments on In Vitro Rumen Methane Production and Fermentation

Rumen fluid for the initial screening experiment and the follow-up fermentation balance experiment was collected on the day of each experiment at approximately 10:00 AM from a single cannulated Jersey cow grazing the same bermudagrass pasture and allowed ad libitum access to water over the course of the two experimental collection periods. Rearing, care and use of the cannulated donor cow was approved by the USDA/ARS Southern Plains Research Center’s Animal Care and Use. The rumen fluid was strained upon collection through a nylon paint strainer [30] into a 500 mL container until full and then immediately capped to minimize oxygen exposure. The strained rumen fluid was returned to the laboratory within 30 min of collection. Upon arrival to the laboratory the fluid was anaerobically distributed (10 mL per tube) while under a continuous flow of 100% carbon dioxide to 18 × 150 mm crimp top culture tubes. The culture tubes were preloaded with small volumes (<0.5 mL) of stock concentrations of candidate modulators (*n* = 3/treatment concentration) as described above, except the 3 mM L-cysteinesulfinic acid treatment was omitted during the screening study due to too little chemical remaining and, based on evidence of effectiveness in the pure culture and screening study, only ethyl nitroacetate and α-lipoic acid were tested in the fermentation balance study. Upon addition of rumen fluid to the culture tubes, the tubes were immediately closed with rubber stoppers and crimped with aluminum seals. Alfalfa, 0.2 g, ground to pass through a 4 mm Willey Mill screen was included as the sole dietary substrate to simulate daily intake of dietary forage. The pH of unused rumen fluid, measured after using an Orion 2 Star pH Benchtop meter (Thermo Electron Corporation, Beverly, MA, USA), for the follow up study was 6.60, pH was not measured during the initial study. The pH was not measured at the end of the 24 h incubations. Tubes were incubated upright without agitation at 39 °C. Untreated controls and treatments were incubated in triplicate during each experiment.

### 2.3. Analytical

After 24 h incubation, gas composition in the headspace gas of each culture was determined by gas chromatography [31]. Briefly, 1 mL of atmosphere from the headspace of each tube was collected via a 1-mL glass syringe and injected into a Gow-Mac series 580 gas chromatograph (Gow-Mac Instrument, Bridgewater, NJ, USA) equipped with a HaySep Q column heated to 60 °C and operated with Argon as the carrier gas flowing at 25 mL/min. Methane and hydrogen were measured with a thermal conductivity detector. Gas volumes in the incubation tubes were measured via insertion of a 30 cc air-tight glass syringe fitted with an 18-gauge needle through the stopper of each tube and recording volume displacement. Molar concentrations of hydrogen and methane were calculated using the Idea Gas Laws and are expressed as µmol/mL of incubation fluid. For incubations in the follow-up experiment, volatile fatty acids were measured in fluid samples collected after 0 and 24 h incubation by gas chromatography [32,33]. Values reported are net amounts produced and were calculated as the difference between concentrations measured in fluid samples collected after 24 h incubation minus initial concentrations. Stoichiometric estimates of amounts of reducing equivalents produced and consumed during incubation of mixed populations of ruminal microbes were based on fermentation balances reported by Ungerfeld et al. [34] except omitting potential contributions of ethanol, lactate and ammonia which were not measured in the present study. Accordingly, amounts of reducing equivalents produced, expressed as µmol hydrogen/mL incubation fluid, were calculated as the sum of (2 acetate) + (1 propionate) + (4 butyrate) + (3 valerate). Amounts of reducing equivalents consumed were calculated as (2 propionate) + (2 butyrate) + (4 valerate) + (4 methane) + (1 hydrogen). Amounts of hexose fermented were calculated as the sum of ½ acetate + propionate + butyrate + valerate + valerate and fermentation efficiency was calculated as (0.62 acetate + 1.09 propionate + 0.78 butyrate) ÷ (acetate + propionate + butyrate) ∗ 100 and is based on the heats of glucose and the respective acids [35].

### 2.4. Statistics

All measures were assessed for normal distribution. If they were not normally distributed, natural log transformation was applied to the measure. All analyses were performed using JMP [36], with the Least Square Fit model specification. Two-way factorial analysis of variance were calculated for each measure and for the main effects of Modulator, Concentration and their interaction for the pure culture experiment. For the unbalanced initial screening study and the follow-up fermentation balance study with mixed rumen culture experiment, the model fixed effects were the main effects of modulator and concentration as well as their interaction. Post hoc multiple comparisons of the interaction effect were determined with the LSMeans Differences with Tukey’s Honestly Significant Difference calculation.

## 3. Results

### 3.1. Antimicrobial Effects on E. coli and Salmonella

**Figure 1:**
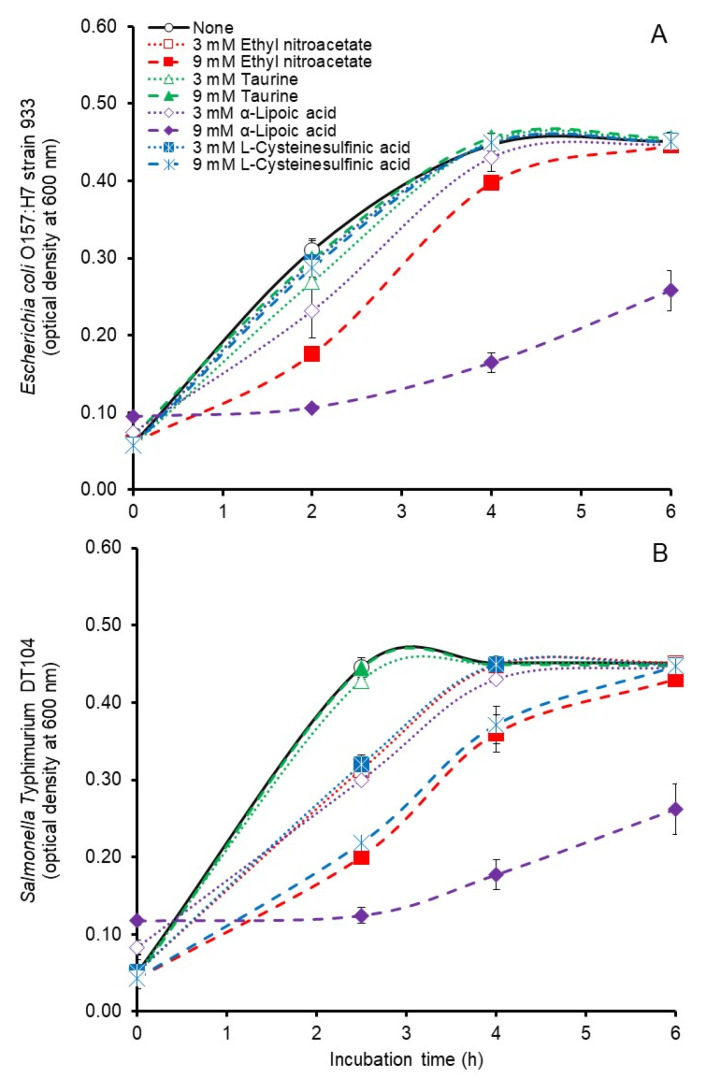
Growth curves of *Escherichia coli* strain 933 (**A**) and *Salmonella* Typhimurium DT104 (**B**) during anaerobic (100% nitrogen gas phase) pure culture in 0.1 M phosphate buffered tryptic soy broth (pH 6.7) supplemented without or with 3 or 9 mM ethyl nitroacetate, taurine, α-lipoic acid or L-cysteinesulfinic acid. Values at each time point are the means ± standard deviations from *n* = 3 cultures/treatment concentration.

Batch culture growth curves generated during the first 6 h of incubation in the present study (Figure 1A,B) revealed the antimicrobial activity of the potential inhibitors. By 6 h of incubation the cultures had achieved maximum optical density and curves extending beyond 6 h to the end of the 24 h incubation did not change appreciably (not shown) indicating that no inhibitory effect occurred during stationary phase. Comparisons for treatment effects under the conditions of the present study revealed significant (*p* < 0.05), albeit modest, inhibitory effects of 3 or 9 mM ethyl nitroacetate on mean specific growth rates of *E. coli* O157:H7 but only a significant effect (*p* < 0.05) of 9 mM ethyl nitroacetate on the growth rate of *S.* Typhimurium (Table 1). Ethyl nitroacetate at 9 mM, but not at 3 mM, decreased (*p* < 0.05) the maximum optical density at 600 nm achieved by *S*. Typhimurium but neither ethyl nitroacetate concentration inhibited the maximum optical densities achieved by *E. coli* O157:H7 (Table 1). Mean specific growth rates of *E. coli* O157:H7 and *S.* Typhimurium were decreased by both 3 and 9 mM α-lipoic acid although maximum optical densities achieved by these bacteria were decreased only by 9 mM added α-lipoic acid (Table 1). Inhibition of *E. coli* O157:H7 or *S.* Typhimurium was not observed with any of the other compounds (Table 1). Significant effects (*p* < 0.05) of the potential inhibitors were observed on the pH measured at the end of incubation of the *E. coli* O157:H7 cultures but these differences were slight and likely of little physiological consequence in the buffered growth medium (Table 1).

### 3.2. Comparative Effects of Inhibitors on In Vitro Rumen Methane Production and Fermentation

**Figure 2:**
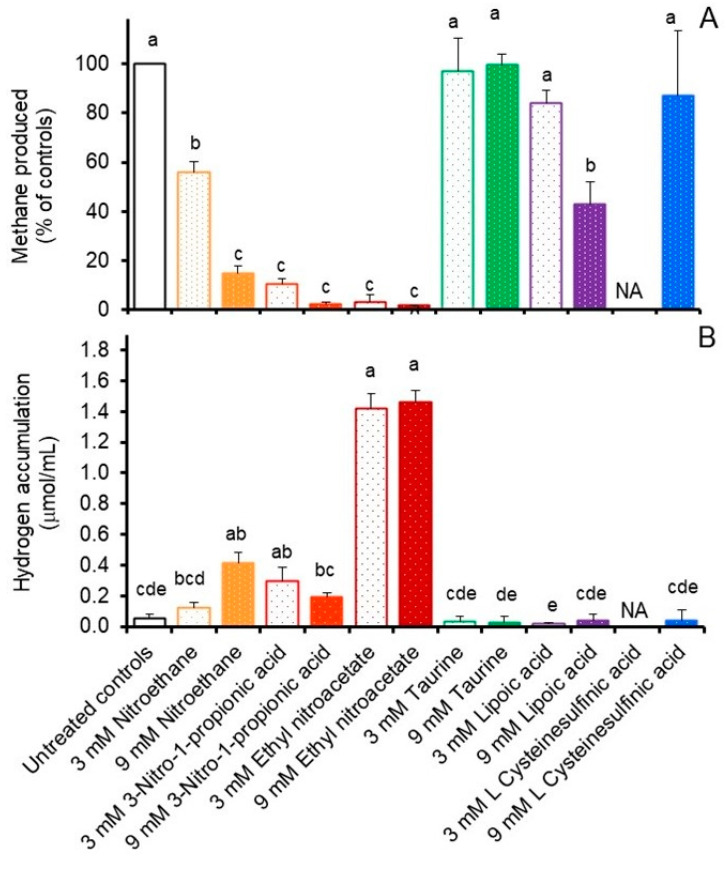
Effect of select compounds on ruminal methane production, expressed as % of controls (**A**), or hydrogen accumulation, expressed as µmol/mL (**B**) after 24 h anaerobic incubation of freshly collected rumen fluid at 39 °C in vitro. Values are least-squares means (±SD) from *n* = 3 cultures/treatment. Standard error of the mean was 4.410 for A and was 0.356 for B. Mean amounts of methane produced for controls ranged from 7.26 to 15.43 and averaged 11.6 ± 5.54 µmol/mL incubation fluid.NA; not available. Means (from *n* = 3 cultures/treatment concentration) with unlike lowercase letters differ (*p* < 0.05) using the LSMeans Differences with a Tukey’s Honestly Significant Difference calculation. Error bars represent standard deviations. NA; not available.

The results from our initial in vitro incubations of freshly collected rumen fluid supplemented without or with the various potential methanogenesis inhibitors are presented in Figure 2. All nitro compound treatments inhibited (*p* < 0.05) methane production, with decreases after 24 h ranging from 43 to 98% compared to untreated controls. Methane production was also decreased by 15 to 57% (*p* < 0.05) during incubation of mixed ruminal populations with 3 or 9 mM α-lipoic acid, respectively, but not by any of the other treatments (Figure 2A). Hydrogen accumulations in these in vitro incubations were affected by treatment (*p* < 0.05), with accumulations being 0.07, 0.36, 0.25, 0.13, 1.36 and 1.40 µmol/mL incubation fluid higher after 24 h in incubations treated with 3 or 9 mM nitroethane, 3-nitro-1-propionic acid or ethyl nitroacetate, respectively, than in untreated controls (0.06 µmol/mL) (Figure 2B). Accumulations of hydrogen in the other treatments were equivalent or lower than those of the controls (not shown) and thus were considered inconsequential (Figure 2B).

The results from a follow-up study characterizing the effects of ethyl nitroacetate, α-lipoic acid or their combination, on in vitro rumen fermentation are presented in Table 2. As observed in the initial screening, ethyl nitroacetate supplemented a 3 or 9 mM inhibited methane production by more than 97% compared to that produced by untreated controls (Table 2). In this experiment, however, methane production was decreased by 47 to 66% in incubations supplemented with 3 or 9 mM α-lipoic acid compared to the controls (Table 2). Hydrogen accumulations were unaffected by treatment (*p* > 0.05) but were as much as 1.09, 1.03 and 1.29 µmol/mL incubation fluid higher in the in vitro rumen incubations treated with ethyl nitroacetate, α-lipoic acid or their combination than in controls (Table 2).

Accumulations of methane and hydrogen in incubations co-treated with ethyl nitroacetate or with α-lipoic acid (each at 3 mM or 9 mM) were similar to those treated solely with equivalent concentrations of ethyl nitroacetate or with α-lipoic acid. While significant (*p* < 0.05), mean accumulations of acetate did not differ appreciably between controls, or incubations treated with ethyl nitroacetate, α-lipoic acid or their combinations regardless of concentration (Table 2). Rumen incubations treated with 3 mM α-lipoic acid had lower butyrate accumulations than incubations treated with 3 mM ethyl nitroacetate but otherwise mean butyrate accumulations were similar between treatments (Table 2). Valerate accumulations were highest in rumen incubations treated with 3 mM ethyl nitroacetate, lowest in incubations treated with the combination of 9 mM ethyl nitroacetate and 9mM α-lipoic acid and intermediate in incubations controls and other treated incubations (Table 2). Accumulations of propionate, isobutyrate, isovalerate and total volatile fatty acids were not significantly affected. Nevertheless, the ratios of acetate to propionate were higher (*p* < 0.05) in the untreated control and 9 mM α-lipoic acid-treated incubations in incubations treated with 3 or 9 mM ethyl nitroacetate, whether alone or in combination with α-lipoic acid, the acetate to propionate ratio being intermediate in the incubations treated solely with 3 mM α-lipoic acid (Table 2). Volatile fatty acid accumulations are presented as mole % in supplementary Table S1.

Stoichiometric estimates of amounts of reducing equivalents produced during the rumen incubations were unaffected by treatment; however, amounts of reducing equivalents consumed decreased in all ethyl nitroacetate-treated incubations, including those combined with α-lipoic acid, when compared to controls, due mainly to the larger decrease in methane produced and the associated amounts of reducing equivalents that otherwise would have been consumed (Table 3). Estimates of amounts of hexose fermented were not affected by treatment; however, fermentation efficiency, calculated as the caloric energy available in amounts such as acetate, propionate and butyrate produced, were higher than controls in incubations treated with 9 mM ethyl nitroacetate as well as co-treatments with either 3 mM or 9 mM of both ethyl nitroacetate and α-lipoic acid (Table 3).

## 4. Discussion

As reviewed by Teng and Kim [37], certain short-chain nitrocompounds have been reported to exert antimicrobial activity against a variety of microbes and their biological activities. While ethyl nitroacetate exhibited quite modest antimicrobial activity against *E. coli* O157:H7 and *S.* Typhimurium, it was very potent at modulating methane production. Likewise, reductions in methane production observed in all the nitrocompound-treated incubations in the present screening study are consistent with observations from earlier studies conducted either with 100% carbon dioxide, as was performed in the present study [13,38,39], or with a 50:50 hydrogen:carbon dioxide as the initial gas phase [13,40,41]. The studies initiated with the 50:50 hydrogen:carbon dioxide gas phase were designed to ensure the provision of a non-limiting amount of reducing substrate. Considering, however, that hydrogen accumulations were higher in all nitro-treated incubations than in untreated controls during the initial mixed rumen population screening study it is reasonable to suspect that the nitrocompounds did not inhibit hydrogenase-producing activity involved in hydrogen evolution. Rather, it seems likely that the nitrocompounds inhibited uptake hydrogenase activity involved in hydrogen consumption, the latter which could restrict hydrogen consumption by methanogens. This hypothesis needs to be tested further; however, considering the diversity of hydrogen- producing and -consuming mechanisms within the rumen [42]. Higher accumulations of hydrogen associated with nitrocompound treatment of mixed populations of ruminal microbes were reported in some of the earlier studies conducted similarly with carbon dioxide as the headspace atmosphere [43,44] but not in another study [13]. With respect to *E. coli* O157:H7 and *S.* Typhimurium, the fermentative conditions of the present study would be expected to favor the production of hydrogen or formate as electron sink products which as indicated above may not be inhibited by the nitrocompounds. Conversely, it may be worthwhile in future studies to test the antimicrobial effects of the nitrocompounds when in the presence of hydrogen- and formate-consuming anaerobic electron acceptors such as nitrate, fumarate or sulfate. This may make the pathogens more susceptible, if as indicated above, the nitrocompounds inhibit uptake hydrogenase or formate-hydrogen lyase activity. In support of this hypothesis, Anderson et al. observed > 4 log_10_ colony forming unit decreases in *E. coli* and *S.* Typhimurium populations during in vitro incubation with pig gut contents supplemented with nitrate and either 2-nitroethanol or 2-nitro-1-propanol when compared to controls supplemented without or with nitrate alone [3].

Evidence reported in the earlier work indicated that some nitrocompounds may act both as direct inhibitors against rumen methanogens and as alternative electron acceptors [13,39], however, based on the low recovery of reducing equivalents in the present study it is unlikely that appreciable amounts of ethyl nitroacetate were reduced here. The accumulation of hydrogen, or contrastingly its microbial consumption, in nitrocompound-treated incubations may be influenced by the presence and activity of a hydrogen-oxidizing, nitro-compound-metabolizing bacterium, *Denitrobacterium detoxificans* [39,44]. This bacterium is normally present at less than 10^3^ cells/mL in rumen populations having no prior exposure to the nitro compounds and although its numbers can be dramatically increased to >10^6^ cells/mL in populations adapted to the nitro compounds, likely, this had not occurred in the present incubations [45]. Accordingly, the microbial reduction of ethyl nitroacetate may require more time to achieve the enrichment of sufficient numbers of *D. detoxificans*. This possibility warrants further study.

Whereas antimicrobial activities of organosulfur compounds such as including sulfonates and sulfinic acids such as taurine and L-cysteinesulfinic acid have been reported [24,25,26,27], little-to-no inhibitory activity against either *E. coli* O157:H7 or *S.* Typhimurium DT104 of these compounds was observed in the present study. This is not surprising, however, as sulfonates and sulfinic acids are recognized more importantly for their antioxidant and anti-inflammatory activity than for their antibacterial activity. However, as discussed below, taurine and L-cysteinesulfinic acid may have been metabolized rapidly enough to preempt potential antibacterial activity. On the other hand, α-lipoic acid is known to function as an important co-factor supporting one carbon compound metabolism in aerobically grown cells [46] and reports of its antimicrobial activity are rare [27]. Consequently, the appreciable antimicrobial activity of α-lipoic acid against *E. coli* O157:H7 and *S.* Typhimurium DT104 observed in the present study was unexpected and this aspect also warrants further study, particularly considering a recent report of α-lipoic acid’s enhancing effect of a natural antimicrobial peptide against methicillin-resistant *Staphylococcus aureus* [47].

In the case of the organosulfur compounds, their metabolism in gut ecosystems also involves reductive processes potentially consuming up to 2 to 6 electrons for their reduction to sulfides that otherwise could be used to reduce carbon dioxide [48]. It is unlikely appreciable reduction of these compounds occurred, at least with taurine and L-cysteinesulfinic acid, as these were not effective in reducing methane production in the present study. Zhang et al. [49], recently reported that the administration of taurine at 0.6 mg/L (the equivalent of 5.3 µmol/mL) decreased methane production by rumen microbes cultured in vitro by 22% [49]. However, taurine was found to be highly degradable by the rumen microbes, with up to 99% being degraded within 2 h [49], which may explain the lack of anti-methane activity in the present study. Stoichiometrically, treatments supplying 9 µmol/mL of the organosulfur compounds could be expected to potentially consume between 18 to 54 electrons (the equivalent of 9 to 27 µmol hydrogen/mL) at the cost of 2.25 to 6.75 µmol methane/mL incubation fluid. In the case of disulfide bond-containing α-lipoic acid, methane production was decreased by 47 to 66% compared to untreated controls but it is unclear as to the mechanism responsible for this decrease. While not necessarily significant, volatile fatty acid accumulations in incubations treated with α-lipoic acid alone or co-treated with α-lipoic acid and ethyl nitroacetate were appreciably lower than in nontreated incubations or those treated solely with ethyl nitroacetate (Table 2). This suggests, but does not prove, that α-lipoic acid addition may have inhibited hexose fermentation, which while not significantly different between treatments was numerically lower in incubations containing α-lipoic acid than those not containing α-lipoic acid (Table 2). Considering that α-lipoic acid, which has a pka of 5.4, is almost as acidic as lactic acid, which has a pka of 5.0, it is possible that the effect of α-lipoic acid on fermentation –as well, as the observed methane-inhibiting activity could be related to its effect on pH. The pH effect was not measured in the present incubations with the mixed populations of rumen microbes. Alternatively, some of the α-lipoic acid may have been biologically reduced to dihydrolipoic acid thereby consuming up to 2 electrons per molecule reduced which could contribute to some of the α-lipoic acid-associated methane inhibition [50,51]. This possibility, however, is not readily discernable in the present experiment. A number of oxidized sulfur-containing compounds are known to be metabolized by rumen or other gut microbes and in some cases, their supplementation to ruminant diets has resulted in improved animal performance [49,52,53,54,55]. Mechanistically, the oxidized sulfur compounds can serve as substrates for the biosynthesis of sulfur-containing amino acids as well as other important nutrients, many of which may be limiting in ruminant diets [52,56]. Additionally, Mazumder et al. [57] reported the metabolism of cysteine and thiosulfate by *Methanosarcina barkeri* via cysteine desulfohydrase and thiosulfate reductase to yield hydrogen sulfide as an end product. Similarly, sulfate metabolizing *Desulfovibrio* spp. can reduce cysteate and/or isethionate and *Bilophila wadsworthia* can degrade taurine [53,58]. Sulfide, produced as a reduced product, can be toxic to animals at high enough concentrations, and thus, dietary recommendations for cattle suggest sulfur intake should not exceed 0.40% of the diet [56,59].

In summary, the results from the present study provide new information on the antimicrobial effects of a selection of oxidized nitro- and sulfur-containing compounds against the foodborne pathogens, *E. coli* O157:H7 and *S.* Typhimurium DT104, with modest activity observed for ethyl nitroaetate and more potent antimicrobial activity observed with α-lipoic acid. Considering that fecal-oral transmission is recognized as the major dissemination route of these foodborne pathogens between animals, the development of feed additive technologies to reduce the gastrointestinal survival of the pathogens is consistent with multi-hurdle feed to food pathogen control strategies. Thus, while ethyl nitroacetate exhibited only modest activity against *E. coli* O157:H7 and *S.* Typhimurium under conditions of the present, it was by far the most potent anti-methanogenic of the compounds tested in this study. The results from the present study further revealed an antimicrobial and anti-methanogenic effect of α-lipoic acid. Ultimately, further research examining the potential of these as well other potential antimicrobials and anti-methanogenic compounds, whether alone or in combination, may yield efficacious technologies able to reduce the carriage of foodborne pathogens while concurrently mitigating ruminal methane emissions, thereby helping livestock producers more cleanly supply microbiologically safe meat and milk to consumers.

## Figures and Tables

**Table 1:** Effect of ethyl nitroacetate, taurine, α-lipoic acid or L-cysteinesulfinic acid on mean specific growth rates and maximum optical densities achieved during anaerobic growth of pure cultures of *Escherichia coli* O157:H7 strain 933 and *Salmonella* Typhimurium DT104 in 0.1 M phosphate buffered (pH 6.7) tryptic soy broth under 100% nitrogen.

	None	Ethyl Nitroacetate		Taurine		α-Lipoic Acid		L-Cysteine- Sulfinic Acid			
		3 mM	9 mM	3 mM	9 mM	3 mM	9 mM	3 mM	9 mM	*p*	SEM
Growth rate (h^−1^)											
*E. coli* O157:H7 933	0.823 ^a^	0.639 ^bc^	0.527 ^c^	0.777 ^a^	0.740 ^ab^	0.568 ^c^	0.224 ^d^	0.809 ^a^	0.809 ^a^	<0.0001	0.033
*S.* Typhimurium DT104	0.886 ^a^	0.744 ^ab^	0.656 ^bc^	0.872 ^a^	0.920 ^a^	0.522 ^c^	0.195 ^d^	0.928 ^a^	0.888 ^a^	<0.0001	0.048
Maximum OD (600 nm)											
*E. coli* O157:H7 933	0.448 ^a^	0.454 ^a^	0.447 ^a^	0.452 ^a^	0.456 ^a^	0.448 ^a^	0.337 ^b^	0.449 ^a^	0.451 ^a^	<0.0001	0.006
*S.* Typhimurium DT104	0.451 ^a^	0.451 ^a^	0.430 ^b^	0.451 ^a^	0.449 ^a^	0.445 ^a^	0.262 ^b^	0.449 ^a^	0.448 ^a^	<0.0001	0.006
Ending pH											
*E. coli* O157:H7 933	6.45 ^b^	6.48 ^ab^	6.46 ^ab^	6.47 ^ab^	6.48 ^a^	6.49 ^a^	6.47 ^ab^	6.45 ^b^	6.40 ^c^	<0.0001	0.006

^a,b,c,d^ Means, from *n* = 3 cultures/treatment concentration, within rows with unlike letter superscripts differ (*p* < 0.05) using the LSMeans Differences with a Tukey’s Honestly Significant Difference calculation.

**Table 2:** Effects of ethyl nitroacetate, α-lipoic acid or their combination on rumen fermentation characteristics after 24 h anaerobic incubation.

	None	Ethyl Nitracetate		α-Lipoic Acid		Both at			
Measured Variable ^1^		3 mM	9 mM	3 mM	9 mM	3 mM	9 mM	*p*	SEM
Total gas (mL)	6.8	3.5	4.2	4.2	5.2	5.2	3.8	0.8123	1.708
Hydrogen (µmol/mL)	0.14	1.23	0.66	1.17	0.23	1.43	0.84	0.2327	0.442
Methane (µmol/mL)	16.36 ^a^	0.53 ^b^	0.28 ^b^	5.63 ^b^	8.71 ^ab^	0.68 ^b^	0.40 ^b^	0.0006	1.928
Total acids (µmol/mL)	51.29	56.10	49.52	30.34	45.70	30.38	41.82	0.0664	6.680
Acetate (µmol/mL)	33.19 ^a^	33.04 ^a^	27.09 ^ab^	18.67 ^ab^	29.54 ^a^	16.13 ^ab^	23.51 ^ab^	0.0472	4.227
Propionate (µmol/mL)	11.85	14.90	16.30	7.90	10.17	9.43	12.42	0.0657	1.905
Butyrate (µmol/mL)	4.68 ^ab^	6.19 ^a^	4.83 ^ab^	2.78 ^b^	4.35 ^ab^	3.62 ^ab^	4.57 ^ab^	0.0257	0.596
Valerate (µmol/mL)	0.94 ^ab^	1.17 ^a^	0.72 ^bc^	0.70 ^bc^	0.94 ^ab^	0.82 ^bc^	0.56 ^c^	0.0012	0.073
Isobutyrate (µmol/mL)	0.27	0.34	0.24	0.12	0.28	0.15	0.28	0.0650	0.052
Isovalerate (µmol/mL)	0.35	0.46	0.33	0.18	0.41	0.23	0.47	0.0620	0.070
Acetate to propionate ratio	2.80 ^a^	2.22 ^bcd^	1.72 ^cd^	2.36 ^ab^	2.88 ^a^	1.66 ^d^	1.88 ^bcd^	0.0001	0.137

^1^ Per mL of incubation fluid; ^a,b,c,d^ Means, from *n* = 3 cultures/treatment concentration, within rows with unlike letter superscripts differ (*p* < 0.05) using the LSMeans Differences with a Tukey’s Honestly Significant Difference calculation.

**Table 3:** Effects of ethyl nitroacetate, α-lipoic acid or their combination on stoichiometric estimates of hydrogen balance, amounts of hexose fermented and fermentation efficiencies after 24 h anaerobic incubation.

	None	Ethyl Nitracetate		α-Lipoic Acid		Both at			
Stoichiometric Estimate		3 mM	9 mM	3 mM	9 mM	3 mM	9 mM	*p*	SEM
e^−^ produced (µmol H_2_/mL) ^1^	99.79	109.28	91.97	58.46	89.48	58.65	79.41	0.0562	12.646
e^−^ consumed (µmol H_2_/mL) ^1^	99.61 ^a^	46.71 ^bc^	44.75 ^bc^	45.76^bc^	65.03 ^ab^	30.06 ^c^	37.00 ^bc^	0.0040	9.203
Observed e^−^ recovery (%)	101.58 ^a^	42.86 ^b^	48.26 ^b^	78.03^ab^	73.40 ^ab^	55.05 ^b^	46.97 ^b^	0.0068	9.208
Theoretical e^−^ recovery (%)	101.58	51.13	78.36	83.21	84.22	77.17	93.17	0.0734	10.764
Hexose fermented (µmol/mL) ^2^	28.14	31.34	27.25	16.76	25.15	17.27	23.10	0.0614	3.601
Fermentation efficiency (%) ^2^	74.72^c^	76.77 ^bc^	79.26 ^ab^	76.19 ^bc^	74.52 ^c^	79.59 ^a^	78.28 ^ab^	0.0010	0.728

^1^ e^−^; electron equivalents, expressed as µmol hydrogen (H_2_)/mL incubation fluid. Amounts of reducing equivalents, expressed as µmol hydrogen/mL incubation fluid, were calculated as the sum of (2 acetate) + (1 propionate) + (4 butyrate) + (3 valerate). Amounts of reducing equivalents consumed were calculated as (2 propionate) + (2 butyrate) + (4 valerate) + (4 methane) + (1 hydrogen) [28]; ^2^ Amounts of hexose fermented were calculated as the sum of ½ acetate + ½ propionate + butyrate + valerate + valerate and fermentation efficiency was calculated as (0.62 acetate + 1.09 propionate + 0.78 butyrate) ÷ (acetate + propionate + butyrate) ∗ 100 and is based on the heats of glucose and the respective acids [29]; ^a,b,c^ Means, from n = 3 cultures/treatment concentration, within rows with unlike letter superscripts differ (*p* < 0.05) using the LSMeans Differences with a Tukey’s Honestly Significant Difference calculation.

## Data Availability

Data for this manuscript are available from the corresponding author, [R.C.A.], upon request.

## Supplementary Materials

The following supporting information can be downloaded at: https://www.mdpi.com/article/10.3390/microorganisms12010034/s1, Table S1: Effects of ethyl nitroacetate, α-lipoic acid or their combination on rumen mol% volatile fatty acid concentrations.
