# Supplementary material for: Assessment of Potential Anti-Methanogenic and Antimicrobial Activity of Ethyl Nitroacetate, α-Lipoic Acid, Taurine and L-Cysteinesulfinic Acid In Vitro
Source: Microorganisms. 2023 Dec 23;12(1):34. doi: 10.3390/microorganisms12010034 (PMC10819541; doi:10.3390/microorganisms12010034)
Supplement: Supplementary file 1 [file microorganisms-12-00034-s001.zip › microorganisms-2721691-supplementary.pdf]

Supplemental Table S1. Effects of ethyl nitroacetate, α-lipoic acid or their combination on rumen mol% volatile fatty acid concentrations.

Measured variable	None	Ethyl nitracetate 3 mM	9 mM	α-Lipoic acid 3 mM	9 mM	Both at 3 mM	9 mM	*P*	SEM
Total acids (μmol/mL)[1]	51.29	56.10	49.52	30.34	45.70	30.40	41.82	0.0664	6.680
Mol%									
Acetate	64.69[a]	58.87[ab]	54.93[bc]	61.47[ab]	64.46[a]	52.08[c]	56.08[bc]	0.0004	1.539
Propionate	23.12[b]	26.57[ab]	32.33[a]	26.09[ab]	22.46[b]	31.79[a]	29.82[ab]	0.0020	1.45
Butyrate	9.12[b]	11.05[ab]	9.98[b]	9.16[b]	9.47[b]	12.10[a]	10.95[ab]	0.0040	0.488
Valerate	1.85[b]	2.10[ab]	1.53[b]	2.31[ab]	2.12[ab]	2.63[a]	1.37[b]	0.0095	0.231
Isobutyrate	0.52	0.60	0.51	0.38	0.60	0.46	0.67	0.0675	0.060
Isovalerate	0.69[b]	0.82[ab]	0.71[b]	0.59[b]	0.88[ab]	0.74[b]	1.12[a]	0.0167	0.081

[1]Per mL of incubation incubation fluid.

[a,b,c,d]Means within rows with unlike superscripts differ ($P < 0.05$) using an LSMeans Differences with a Tukey's Honestly Significant Difference calculation.
